# Supplementary material for: Commercial Yeast Strains Expressing Polygalacturonase and Glucanase Unravel the Cell Walls of Chardonnay Grape Pomace
Source: Biology (Basel). 2022 Apr 26;11(5):664. doi: 10.3390/biology11050664 (PMC9137979; doi:10.3390/biology11050664)
Supplement: Supplementary file 1 [file biology-11-00664-s001.zip › biology-1678086-supplementary.pdf]

Supplementary material

Table S1. Strains and plasmids used and created in this study

STRAIN OR PLASMID	DESCRIPTION	SOURCE OR REFERENCE
**Bacterial strain**		
E. coli DH5a	F′ø 80ΔlacZΔM15Δ(lacZYA-*arg*F)U169 *deoR reA1 hsdR17* (r_{K}^{-} m_{K}^{+}) *supE44*l^{-} *thi1 gyr A96 relA1/F′proABlacl*^{q}ZΔM15,*zzf::Tn5*[*Km*^{r}]	[a]GIBCO-BRL/ Life Technologies
**Plasmids**		
pGEM®-T Easy	*Ap*^{R} Δ*lacZ*	[b]Promega Corporation
pUT332	*Ap*^{R} *Tn5 ble*	[38]
pUG6	*Ap*^{R} *loxP* $TEF2_{P}$ *kan*^{r} $TEF2_{T}$ *loxP*	[39]
pDRIVE	*Ap*^{R} *kan*^{r}Δ*lacZ*	[c]Qiagen GmbH
pAR5	*Ap*^{R}*Gt*^{R}*LEU2* $ADC1_{P}$ $MFa1_{S}$ *end1* $TRP5_{T}$ $ADC1_{P}$ $MFa1_{S}$ *pelE* $TRP5_{T}$ $ADC1_{P}$ $MFa1_{S}$ *peh1* $TRP5_{T}$	[40]
pPOF1	PCR product POF1 cloned into pGEM®-T Easy	This study
pPOF1b	pPOF1 with the *Sal*I site in the [d]MCS destroyed and a new *Sal*I site introduced within the POF1 ORF	This study
pPOF1b KMX	[e]KanMX^{R} cloned in the *Bam*HI and *Sal*I sitesof pPOF1b	This study
pMPOF1	PCR product MPOF1 cloned into pGEM®-T Easy	This study
pMPOF1b	pMPOF1 with the *Sal*I site in the MCS destroyed and a new *Sal*I site introduced within the MPOF1 ORF	This study
pMPOF1bEND1	pGEM®-T Easy with $ADC1_{P}$ $MFa1_{S}$ *end1* $TRP5_{T}$ cloned between MPOF1 homologous flanks	This study
**Yeast strains**		
VIN13	*Saccharomyces cerevisiae*, Commercial diploid strain	[f]Anchor yeast.
V13 KMX	VIN13 pof1::KanMX^{R}	This study
V13 END1	VIN13 pof1:: $ADC1_{P}$ $MFa1_{S}$ *end1* $TRP5_{T}$	This study
PR7	VIN13 x *Saccharomyces paradoxus* RO88 hybrid	[25]
PR7 KMX	PR7 pof1::KanMX^{R}	This study
PR7 END1	PR7 pof1:: $ADC1_{P}$ $MFa1_{S}$ *end1* $TRP5_{T}$	This study

[a]GIBCO/Bethesda Research Laboratories, Life Technologies Ltd., USA; [b]Promega corporation, USA; [c]QIAGEN, Netherland; [d]MSC, Multiple cloning site; [e]KanMX^{R}, geneticin resistance expression cassette; [f]Anchor yeast, South Africa

Table S2. Primers designed for this study and PCR programs used

Primer name	Sequence 5'- 3	Restriction sites included
POF1-L	AGGCCTGCCCGGGCACCCATTTTTAAGATTGGTG	*Stu*I, *Srf* I
POF1-R	AGGCCTGCCCGGGCTTGGTGATGTAATAATGTCAAG	*Stu*I, *Srf* I
POF1-M	GTACGGATCCACGTGTCGACTCGTTGGGAAGGAATAAA	*Bam*HI, *Sal*I
MaxiPOF1-L	TCGCGAGCCCGGGCCCATGGATGTTCTATGAATG	*Nru* I, *Srf* I
MaxiPOF1-R	TCGCGAGCCCGGGCCTTACACTCCAGCAAATAATC	*Nru* I, *Srf* I
exPOF1-L	AATAGCCCGACTCCGTAG	
ENDSEQ	GCCTTCAGCTTCTGTAGACT	
KanMX-Rp	CTCCATGGTGGGCAGATGATGTCGAGG	
**PCR program number**	**Amplification cycle***	
1	94 °C, 30 s; 53 °C, 30 s; 72 °C, 90 s	
2	45 °C, 30 s; 55 °C, 30 s; 72 °C, 140 s	
3	94 °C, 30 s; 55 °C, 30 s; 72 °C, 34 s	
4	94 °C, 30 s; 52 °C, 30 s; 72 °C, 240 s	
5	94 °C, 30 s; 53 °C, 30 s; 72 °C, 150 s	

* All PCR programs had an initial denaturation step at 94 °C for 5 min and a final elongation step at 72 °C for 7 min

Table S3. Monoclonal antibodies (mAbs) and carbohydrate binding modules (CBMs) used in this study.

**Monoclonal antibody**	**Reference**
HG partially/de-esterified (mAb JIM5)	[41]
HG partially esterified (mAb JIM7)	[41]
HG partially/de-esterified (mAb LM18)	[41]
HG partially/de-esterified (mAb LM19)	[41]
HG partially esterified (mAb LM20)	[41]
HG ±30 contiguous unmethylesterified GalA[a] units (mAb PAM1)	[42]
HG Ca^{2+} dimers (mAb 2F4)	[43]
RG-I, 6 unbranched disaccharide (mAb INRA-RU1)	[44]
RG-I, 2 unbranched disaccharide (mAb INRA-RU2)	[44]
α-1,4-D-galactan (mAb LM5)	[45]
α -1,5-L-arabinan (mAb LM6)	[46]
Linearised α -1,5-L-arabinan (mAb LM13)	[47]
α -1,4-D-(galacto)(gluco)mannan (mAb LM21)	[48]
α -1,4-D-(gluco)mannan (mAb LM22)	[48]
α -1,3-D-glucan (mAb BS-400-2)	[47]
Xyloglucan (XXXG motif) (mAb LM15)	[48]
Xyloglucan (XLLG oligosaccharide) (mAb LM24)	[49]
Xyloglucan (mAb LM25)	[49]
α -1,4-D-Xylan (mAb LM10)	[50]
α -1,4-D-Xylan d/arabinoxylan (mAb LM11)	[50]
Celulose (crystalline) (mAb CBM3a)	[51]
Extensin (mAb LM1)	[52]
Extensin (mAb JIM11)	[53]
Extensin (mAb JIM20)	[53]
AGP (mAb JIM8)	[54]
AGP (mAb JIM13)	[55]
AGP (mAb LM14)	[47]
AGP, α -linked GlcA[b] (mAb LM2)	[55]

[a]galacturonic acid; [b]GlcA, glucuronic acid;

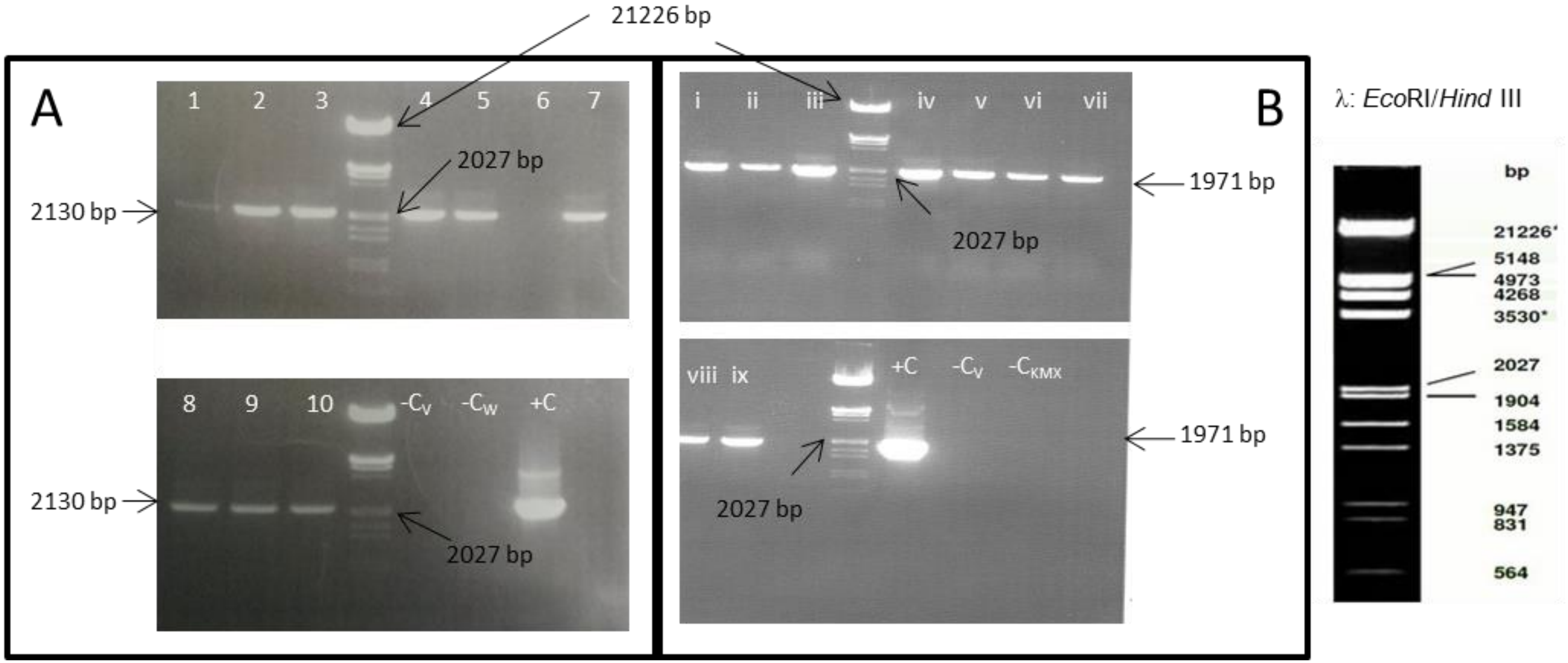

Figure S1 (A). Proof of integration of the kan^r integration cassette into the VIN13 genome with ten putative transformants of V13 KMX. A PCR product of 2130bp with primer pair POF1-L and KanMX-Rp were obtained for nine of the ten transformants. Genomic DNA of VIN13 (-C_V) and MilliQ water (-C_W) was used as a negative control while pPOF1bKMX DNA was used as positive control (+C) for the PCR reaction. **(B).** A PCR product of 1971 bp indicated that the correct integration of the END1 integration cassette took place at the *POF1* locus of VIN13. On the gel the V13 END1 yeast transformants were in lane i, transformant # 1.8; lane ii, 1.26; iii, 29.30; iv, 36.31; v, 45.18; vi, 46.29; vii, 47.1; viii, 52.14; ix, 34.3. Plasmid pMPOF1bEND1 was used a positive control (+C), and negative controls were VIN13 (-C_V) and V13 KMX (-C_{KMX}). The DNA molecular weight marker was Lambda DNA cut with restriction enzymes *Eco*RI and *Hind*III.

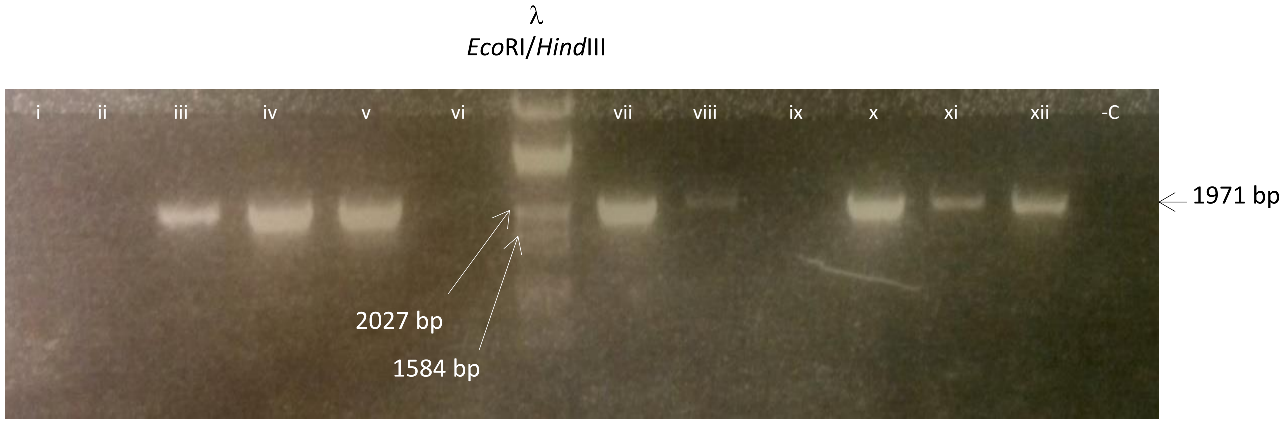

Figure S2. PR7 END1 recombinant transformants identified with PCR using primer pair exPOF1-L and ENDSEQ. A product of 1971 bp indicates that the END1 integration cassette was integrated at the correct position on the PR7 genome. Lane i, transformant # 2.31; ii, 3.1; iii, **3.28**; iv, **4.17**, v, **4.31**; vi, 4.40; DNA molecular weight marker (λ: *Eco*RI/*Hind*III), vii, **4.51;** viii, 6.44; ix, 7.17; x, **10.10**; xi, **10.19**; xii, **10.41.** The transformants that gave the correct PCR product are shown in bold letters. Genomic DNA of PR7 was used as negative control (-C).

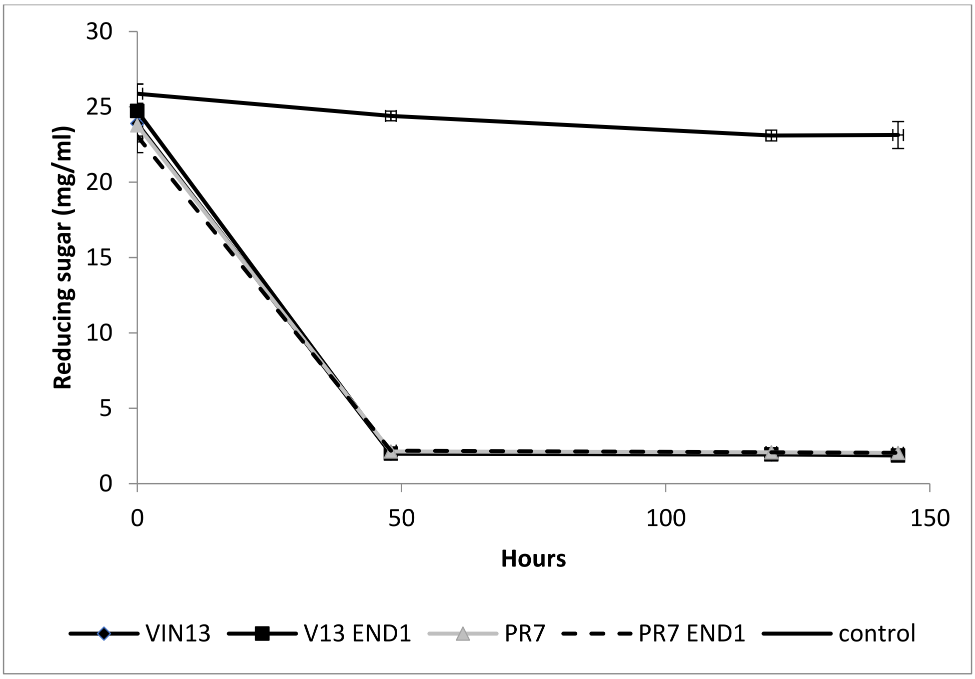

Figure S3. Reducing sugars in the supernatant of the fermentations with the yeast strains VIN13, VIN13 END1, PR7 and PR7 END1 with Chardonnay pomace suspension as substrate. The values are the average of four biological repeats.

References

25. Mocke, B. The Breeding of Yeast Strains for Novel Oenological Outcomes. Master's Thesis, Stellenbosch University, Stellenbosch, South Africa, 2005.
38. Gatignol, A.; Dassain, M.; Tiraby, G. Cloning of *Saccharomyces cerevisiae* promoters using a probe vector based on phleomycin resistance. Gene 1990, 91, 35–41.
39. Güldener, U.; Heck, S.; Fielder, T.; Beinhauer, J.; Hegemann, J.H. A new efficient gene disruption cassette for repeated use in budding yeast. Nucleic Acids Res. 1996, 24, 2519–2524.
40. van Rensburg, P.; Van Zyl, W.H.; Pretorius, I.S. Expression of the *Butyrivibrio fibrisolvens* endo-beta-1,4-glucanase gene together with the *Erwinia* pectate lyase and polygalacturonase genes in *Saccharomyces cerevisiae*. Curr. Genet. 1994, 27, 17–22.
41. Verhertbruggen, Y.; Marcus, S.E.; Haeger, A.; Ordaz-Ortiz, J.J.; Knox, J.P. An extended set of monoclonal antibodies to pectic homogalacturonan. Carbohydr. Res. 2009, 344, 1858–1862. https://doi.org/10.1016/j.carres.2008.11.010.
42. Willats, W.G.T.; Gilmartin, P.M.; Mikkelsen, J.D.; Knox, J.P. Cell wall antibodies without immunization : Generation and use of de-esterified homogalacturonan block-specific antibodies from a naive phage display library. Plant J. 1999, 18, 57–65.
43. Liners, F.; Letesson, J.-J.; Didembourg, C.; Van Cutsem, P. Monoclonal antibodies against pectin: Recognition of a conformation induced by Calcium. Plant Physiol. 1989, 91, 1419–1424. https://doi.org/10.1104/pp.91.4.1419.
44. Ralet, M.-C.; Tranquet, O.; Poulain, D.; Moïse, A.; Guillon, F. Monoclonal antibodies to rhamnogalacturonan I backbone. Planta 2010, 231, 1373–1383. https://doi.org/10.1007/s00425-010-1116-y.
45. Jones, L.; Seymour, B.; Knox, J.P. Localization of pectic galactan in tomato cell walls using a monoclonal antibody specific to 1,4-B-D-Galactan. Plant Physiol. 1997, 113, 1405–1412.
46. Willats, W.G.T.; Marcus, S.E.; Knox, J.P. Generation of a monoclonal antibody specific to 1,5-a-L-arabinan. Carbohydr. Res. 1998, 308, 149–152.
47. Moller, I.; Marcus, S.E.; Haeger, A.; Verhertbruggen, Y.; Verhoef, R.; Schols, H.; Ulvskov, P.; Mikkelsen, J.D.; Knox, J.P.; Willats, W. High-throughput screening of monoclonal antibodies against plant cell wall glycans

by hierarchical clustering of their carbohydrate microarray binding profiles. Glycoconj. J. 2008, 25, 37–48. https://doi.org/10.1007/s10719-007-9059-7.
48. Marcus, S.E.; Blake, A.W.; Benians, T.A.S.; Lee, K.J.D.; Poyser, C.; Donaldson, L.; Leroux, O.; Rogowski, A.; Petersen, H.L.; Boraston, A.; et al. Restricted access of proteins to mannan polysaccharides in intact plant cell walls. Plant J. 2010, 64, 191–203. https://doi.org/10.1111/j.1365-313X.2010.04319.x.
49. Pedersen, H.L.; Fangel, J.U.; McCleary, B.; Ruzanski, C.; Rydahl, M.G.; Ralet, M.-C.; Farkas, V.; von Schantz, L.; Marcus, S.E.; Andersen, M.C.F.; et al. Versatile high resolution oligosaccharide microarrays for plant glycobiology and cell wall research. J. Biol. Chem. 2012, 287, 39429–39438. https://doi.org/10.1074/jbc.M112.396598.
50. McCartney, L.; Marcus, S.E.; Knox, J.P. Monoclonal antibodies to plant cell wall xylans and arabinoxylans. J. Histochem. Cy-tochem. 2005, 53, 543–546. https://doi.org/10.1369/jhc.4B6578.2005.
51. Blake, A.W.; McCartney, L.; Flint, J.E.; Bolam, D.N.; Boraston, A.B.; Gilbert, H.J.; Knox, J.P. Understanding the biological rationale for the diversity of cellulose-directed carbohydrate-binding modules in prokaryotic enzymes. J. Biol. Chem. 2006, 281, 29321–29329. https://doi.org/10.1074/jbc.M605903200.
52. Neumetzler, L.; Humphrey, T.; Lumba, S.; Snyder, S.; Yeats, T.H.; Usadel, B.; Vasilevski, A.; Patel, J.; Rose, J.K.C.; Persson, S.; et al. The FRIABLE1 gene product affects cell adhesion in *Arabidopsis*. PLoS ONE 2012, 7, e42914. https://doi.org/10.1371/journal.pone.0042914.
53. Smallwood, M.; Beven, A.; Donovan, N.; Neill, S.J.; Peart, J.; Roberts, K.; Knox, J.P. Localization of cell wall proteins in relation to the developmental anatomy of the carrot root apex. Plant J. 1994, 5, 237–246.
54. Pennell, R.; Janniche, L.; Kjellbom, P.; Scofield, G.N.; Peart, J.M. Developmental regulation of a plasma membrane arabino-galactan protein epitope in oilseed rape. Plant Cell 1991, 3, 1317–1326.
55. Yates, E.A.; Valdor, J.F.; Haslam, S.M.; Morris, H.R.; Dell, A.; Mackie, W.; Knox, J.P. Characterization of carbohydrate struc-tural features recognized by anti-arabinogalactan-protein monoclonal antibodies. Glycobiology 1996, 6, 131–139.
